# Characterization of the Cap ‘n’ Collar Isoform C gene in *Spodoptera frugiperda* and its Association with Superoxide Dismutase

**DOI:** 10.3390/insects11040221

**Published:** 2020-04-02

**Authors:** Yiou Pan, Xiaochun Zeng, Shuyuan Wen, Xuemei Liu, Qingli Shang

**Affiliations:** 1 College of Plant Science, Jilin University, Changchun 130062, China; panyo@jlu.edu.cn (Y.P.); zengxc18@mails.jlu.edu.cn (X.Z.); shielawen@163.com (S.W.); liuxm18@mails.jlu.edu.cn (X.L.); 2 School of Agricultural Science, Zhengzhou University, Zhengzhou 450001, China

**Keywords:** CNC-bZIP, superoxide dismutase, transcription regulation, RNAi, *Spodoptera frugiperda*

## Abstract

Nuclear factor erythroid 2 related factor 2 (Nrf2) belongs to the cap ‘n’ collar basic region leucine zipper (CNC-bZIP) transcription factor family, and is activated by diverse oxidants, pro-oxidants, antioxidants, and chemo-preventive agents. Transcriptional regulation of a battery of detoxifying and antioxidant genes by Nrf2 has been shown to be important for protection against oxidative stress or chemically-induced cellular damages. In our research, we cloned the full length CncC gene from the *Spodoptera frugiperda*, named as *Sf*CncC. The cDNA of the *Sf*CncC consists of 2652 nucleotides that include a 2196-nucleotide open reading frame (ORF), encoding 731 amino acid residues, and 239- and 217-bp non-coding regions flanking at the 5’- and 3’-ends of the cDNA, respectively. Sequence analysis indicated *Sf*CncC has the conserved domain (CNC-bZIP domain and a tetrapeptide motif, ETGE) character of Nrf2 and showed high identity compared with the CncC/Nrf2 from other insect and vertebrate species. Over-expression of *Sf*CncC can up-regulate the transcription and activity of the SOD gene in Sf9 cells, and the RNAi of *Sf*CncC in Sf9 cells and larvae of *S. frugiperda* can dramatically reduce the transcriptional level and activity of the SOD gene, as determined by real-time quantitative PCRs. So the *Sf*CncC is involved in the Keap1-Nrf2-ARE pathway, acting the same as the transcriptional factor Nrf2 in vertebrate, and plays a role for host cell defense. The functional characterization of *Sf*CncC provides the fundamental basis for us to further understand the regulatory mechanism of anti-oxidants and anti-xenobiotics in *S. frugiperda.*

## 1. Introduction

Cellular detoxification is crucial for the maintenance of health by providing protection against the daily exposure to various xenobiotics [1,2,3]. This is achieved by regulating the basal and inducible expression of numerous detoxifying and antioxidant genes. Nuclear factor erythroid 2 related factor 2 (Nrf2) belongs to the cap ‘n’ collar basic region leucine zipper (CNC-bZIP) transcription factor family, and is activated by diverse oxidants, pro-oxidants, antioxidants, and chemo-preventive agents [4]. Nrf2 is the central transcriptional factor involved in regulating the expression of antioxidant and phase II metabolizing enzymes important in the protection of cells against oxidative damages caused by electrophiles and reactive oxidants. Nrf2 promotes the expression of various cytoprotective genes by binding to antioxidant responsive elements (ARE) in the 5’-flanking regions of many detoxifying genes in response to xenobiotic and oxidative insults [5,6,7,8]. Under normal situations, the Nrf2 protein is continually degraded in the cytoplasm by an E3 ubiquitin ligase complex containing the regulatory protein Kelch-like ECH-associated protein 1 (Keap1) [9,10,11,12]. Under oxidation stress, cysteine residues of Keap1 will be modified, then after phosphorylation and dissociation from the Keap1, Nrf2 translocates to the nucleus, and, along with small Maf proteins, binds to an antioxidant response element (ARE) in target gene promoters, accelerating their transcription [13,14]. The Keap1-Nrf2-ARE pathway that coordinately up-regulates many protective detoxification and antioxidant genes—including superoxide dismutase (SOD), catalase, several classes of glutathione-S-transferase (GST), and some cytochrome P450 (P450)—can synergistically increase the efficiency of cellular defense system [15,16,17,18,19,20].

A study demonstrated that a parallel signaling pathway to the vertebrate Keap1-Nrf2-ARE cascade also exists in *Drosophila. melanogaster*. The *D*. *melanogaster* proteins Cnc isoform C (CncC) and Keap1 are the authentic homologues of the vertebrate Nrf2 and Keap1, respectively. [21]. Like in vertebrates, the Keap1-Nrf2-ARE signal pathway in the fruit fly is activated by oxidants, induces antioxidant and detoxification responses, and confers increased tolerance to oxidative stress [21]. The fall armyworm, *Spodoptera frugiperda*, is one of the most destructive insect pests on the American continent [22,23]. Larvae of this insect are polyphagous, and among the most damaging pests of at least 30 crops, including maize, sorghum, rice, cotton, sugarcane, soybeans, peanuts, and many forage grasses. Its highly mobile and migratory ability, coupled with resistance to both traditional insecticides and *Bacillus thuringiensis* (Bt) [24,25,26], enable *S. frugiperda* to become more destructive. *S. frugiperda* encounters much stresses in the environment, especially xenobiotic chemicals produced by host plants as the defensive compounds. To defer the cost of counter defense against plant defenses, herbivorous insects have evolved a complex of regulatory machinery, enabling substantial increases of their detoxification and antioxidant activities upon exposure to xenobiotic chemicals. Such xenobiotic chemicals inducibility is essential for insect herbivores to cope with a diversity of xenobiotic chemicals idiosyncratically distributed among potential host plants. In *Tribolium castaneum*, *S. exigua*, and *S. litura*, several GSTs, P450s, and UDP-glycosyltransferases (UGTs) that are responsible for the xenobiotic stresses are also regulated by this mechanism [27,28,29,30,31,32]. Understanding the regulatory cascades will provide us with more insight into the mechanism of the *S. frugiperda* response to xenobiotic stress, and for designing novel and environmentally benign methods to control this pest. To advance understanding of anti-xenobiotic regulatory mechanism in *S. frugiperda*, we cloned and analyzed the *Spodoptera frugiperda* (*Sf*CncC) isoform gene, further analyzed its function by transient over-expression and RNAi.

## 2. Material and Methods

### 2.1. Cell Culture and Insects

Sf9 cells were routinely cultured in SF-900 II serum-free medium (Invitrogen, Carlsbad, CA, USA), supplemented with 10% heat-inactivated fetal bovine serum (FBS, Hyclone-QB perbio, Logan, UT, USA), 50 U/mL penicillin, 50 µg/mL streptomycin, and 12 µg/mL gentamycin (Invitrogen, Carlsbad, CA, USA) at 28 °C.

A laboratory strain of *S. frugiperda* was maintained in an insectary kept at 28 °C with a photoperiod of 16 h light: 8 h dark on a semi-synthetic diet containing wheat germ. Six second instar larvae randomly picked from the colony were individually flash-frozen in liquid nitrogen and stored at −80 °C for subsequent RNA isolation.

### 2.2. Extractions of RNA and Synthesis of cDNA

Total RNA was isolated from cells and larvae using the TRIzol (Invitrogen, Carlsbad, CA, USA) and was treated with DNase (RNase free) to exclude DNA contamination. The RNA was quantified by measuring the absorbance at 260 nm, and the quality was checked by electrophoresis through agarose gel electrophoresis stained with ethidium bromide (EB). First-strand cDNA was synthesized using a superscript III reverse transcriptase (Invitrogen, Carlsbad, CA, USA) with 2 μg total RNA as template and oligo-dT_18_ as primer.

### 2.3. Rapid Amplification of cDNA Ends (RACE)

We successfully cloned a 647 bp cDNA fragment sequence of the *Sf*CncC by homology-based RT-PCR with degenerate primers SfCF (5’-GCCCAGAAGAAGCAYCASMTGTT-3’) and SfCR (5’-CTGGTCCARTTTSCGTTTCCKGC-3’). The RT-PCR amplifications were performed using an *Ex* Taq DNA polymerase (TaKaRa, Dalian, China) with denaturation for 5 min at 94 °C, followed by 35 cycles consisting of 94 °C denaturation for 30 s, 60 °C annealing for 30 s, 72 °C extension for 2 min, and a final 72 °C extension for 10 min. Amplified fragments were purified from agarose gels and ligated into the pGEM-Teasy vector (Promega, Madison, MI, USA), then transformed into the *DH5α* competent cells. The successfully recombinant plasmids were sequenced by Sangon Biotech Co., Ltd. (Shanghai, China).

In order to obtain the complete CncC, rapid amplification of cDNA ends were taken with the GeneRacer™ Kit (Invitrogen, Carlsbad, CA, USA). The synthesized 5’- and 3’-RACE-Ready cDNAs were employed as templates to PCR-amplify the 5’ and 3’ end of *Sf*CncC cDNA. For the 5’RACE, the 5’GeneRace primer supplied by the kit and a gene specific primer Reverse GSP1, (5’-ATCTGTTAGCCGCCGCACCCGTTTGT-3’) were used to perform the first round of PCR. The product was used as template to carry out a secondary PCR using the primer pair of 5’Nest GeneRace and Reverse GSP2 Reverse GSP 2 (5’-ACTTCACAACGCTCGGTCTGCCAACA-3’). For 3’ RACE, the primer pair of 3’GeneRace primer and Forward GSP1, 5’-ACAAACGGGTGCGGCGGCTAACAGAT-3’ were used for the first round PCR, and the primer pair of 3’Nest GeneRace primer and Forward GSP2, 5’-ACTGCCGCAAACGCAAGCTAGACCAG-3’) were used for the secondary PCR. The first round cycling parameters were 5 cycles of 94 °C for 30 s, 72 °C for 3 min, 5 cycles of 94 °C for 30 s, 70 °C for 30 s, 72 °C for 3 min, 25 cycles of 94 °C for 30 s, 68 °C for 30 s, and 72 °C for 3 min, followed by the secondary PCR consisting of 30 cycles of 94 °C for 30 s, 68°C for 30 s, and 72 °C for 3 min. The PCR products were electrophoresed on a 1% agarose gel in 1×TAE buffer. The resultant bands were eluted from the gel using the QIAquick Gel Extraction Kit (Qiagen, Frederick, MD, USA), and then directly cloned into the pGEM-Teasy vector (Promega, Madison, MI, USA) and transformed into *DH5α* competent cells. Two positive clones were sequenced.

The *Sf*CncC fragment (2228bp) containing the full ORF was amplified by using two pairs of CncC specific primers (C1F, 5’-CTCCTTGTGACCACCAGCCTA-3’ and C1R, 5’-TTTATTGCATGGTCTCGAGTCTG-3’, C2F, 5’-CGGAGACAACATGCTTCTTGACGAG-3’, and C2R, 5’-ACTGGTTGTAGTAGGCGGTATGTCACT-3’,). The conditions for the RT-PCR amplifications were denaturation for 5 min at 94 °C, followed by 30 cycles consisting of 94 °C denaturation for 30 s, 60 °C annealing for 30 s, a 72 °C extension for 2 min, and a final 72 °C extension for 10 min. The first round PCR product was used as template in the second round PCR. Amplified fragments were purified from agarose gels and ligated into the pGEM-Teasy vector (Promega, Madison, MI, USA) to become pGEM-*Sf*CncC, then transferred into the *DH5a* competent cells. The successfully recombinant plasmids were sequenced.

### 2.4. Transient Transfection

To express *Sf*CncC or *Sf*CncC RNAi analysis in cells, Sf9 cells seeded onto a 12-well plate (9 × 10 ^5^ cells/well) were transiently transfected using a Cellfectin-II reagent (8 µL per well) with the constructs or dsRNA (2 µg/well). Each treatment included three replicates. After 48 h (transfection with constructs) or 96 h (transfection with dsRNA), the cells were harvested by a 5-min centrifugation at 800 g and stored at −80 °C for RNA isolation.

### 2.5. Overexpression of SfCncC Regulates SOD Transcription in Sf9 Cells

The *Sf*CncC ORF was amplified by PCR with one pair primer (CncC-F, 5’-AGGCGCGCCATGCTTCTTGACGAGGT-3’ and CncC-R, 5’-GTTTAAACTCACTGGTCG TAGCTTTTAGC-3’) from pGEM-*Sf*CncC that contains the whole CncC full ORF sequence, and was cloned into the Asc I and Pme I restriction site of the pAC-V5 modified vector (in which we introduced Asc I into its cloning region) to form a pAC-*Sf*CncC construct. Sf9 cells transfected with the pAC-*Sf*CncC construct were harvested after a 48 h incubation at 28 °C for total RNA isolation and cDNA synthesis. Real-time quantitative PCR was performed by using the following primers to detect the SOD gene (The primers were designed based on the SOD sequence of *S. exigua*, Genbank EU_263634, the PCR product has been sequenced and confirmed to be the SOD gene of *S. frugiperda*. SOD-F, 5’-GTGTGTTCTCAAGGGCGATGT-3’, SOD-R, 5’-ATTACACCACAAGCGATGCGA-3’). The elongation factor 1 alpha (EF-1α) gen (EF-1α-F, 5’-GACAAACGTACCATCGAGAAG-3’, EF-1α-R, 5’-GGTACAGCCTCCTGGAGAGC-3’) was used as an internal reference. Quantitative real time RT-PCR was performed on ABI 7500 (Applied Biosystems, Carlsbad, CA, USA) using an SYBR Premix ExTaq kit (Takara, Shiga, Japan). The experiment was independently conducted three times with different RNA preparations. The reactions were performed in a 25 μL mixture, which contained 24 μL of PCR mixture (SYBR Premix) and 1 μL cDNA (equivalent to 0.05 μg of total RNA). The cycling parameters were 95 °C for 1 min; followed by 40 cycles of 95 °C for 30 s; 60 °C for 30 s; 72 °C for 1 min, and l s at 80.5 °C for plate reading. After the cycling protocol, the final step was applied to all reactions by continuously monitoring fluorescence through the dissociation temperature of the PCR product at a temperature transition rate of 0.1 °C/s to generate a melting curve. Quantification was conducted according to the 2^−ΔCt^ method [33].

### 2.6. RNAi Analysis of SfCncC Regulating SOD Gene Expression in sf9 Cells and Larvae

The 419 bp CncC and 540 bp dsRed DNA fragments were amplified using primer pairs of C-RNAiF (5’-GGAGGTGCAAGACATAATC-3’)/C-RNAiR (5’-TGCGGTTCATCGGATGATC-3’) and R-RNAiF(5’-ATGGATAGCA CTGAGAACGT-3’)/R-RNAiR (5’-GATTGACTTG AACTCCACCA-3’), then cloned into pGEM-Teasy vector. Double-stranded RNA (dsRNA) was synthesized using T7 RiboMAX Express RNAi System (Promega, Madison, MI, USA), following the manufacturer’s instructions. The synthesized dsRNA were dissolved in nuclease-free water, and analyzed on 1.5% agarose gel to determine its quality. The Sf9 cells were transiently transfected with the dsRNA (Cnc dsRNA or DsRed dsRNA) as described above. Total RNA was isolated from cells for cDNA synthesis after a 96 h transfection. Each treatment included three replicates. The second instar larvae were used for dsRNA (2 µg/µL) injection with 1 microgram of the dsRNA into the abdomen by microinjection (WPI, Sutter, USA). Control larvae were injected with equivalent volumes of dsRed dsRNA (2 µg/µL) alone. Total RNA was extracted from these larvae for cDNA synthesis after apply dsRNA for 72 h. Each treatment included three replicates (six larvae were used in each replication). Real-time quantitative PCR was performed to detect the *SOD* gene and *Sf*CncC gene transcription change, as described above. The transcriptional level of the *Sf*CncC gene was detected by using primer pair of SfCF (5’-ACCGCACACTCGAGCCATGC-3’) and SfCR (5’-ACCAGCACCACGTTGCCGTC-3’). The real-time PCR cycling parameters for *Sf*CncC were the same as described above.

### 2.7. SOD Activity Determination

Six larvae (dsRNA injected with Cnc dsRNA or DsRed dsRNA) or cells of three wells (transfected with Cnc dsRNA or DsRed dsRNA) were ground in liquid nitrogen, and then suspended in a 0.9 mL solution containing 10 mM phosphate buffer (pH 7.4). The homogenate was centrifuged at 4 °C, 2500 rpm for 10 min, and the resulting supernatant were used for the determination of total SOD activities, according the instructions of commercial assay kits (Nanjing Jiancheng Bioengineering Institute, Nanjing, China). All the enzymes above were detected in each experimental replicate (n = 3). Total SOD activity was assayed using the xanthine/xanthine oxidase method, based on the production of O^2−^ anions. Each endpoint assay was detected by the red substances of the reaction system by absorbance at 550 nm after reaction. SOD activities are expressed as units per milligrams of protein (U/mg protein).

### 2.8. Sequence and Data Analysis

The DNAMAN 6.0 software was used to predict the putative open reading frame (ORF) of *Sf*CncC. Searches for homologous sequences and the predication of conserved leucine zipper region were performed by using BLAST on NCBI. ClustalW software (www.ebi.ac.uk/Tools/msa/clustalo) was used to perform a multiple sequence alignment prior to phylogenetic analysis. The MEGA 6.0 program (http://www.megasoftware.net/) was used to construct the consensus phylogenetic tree using the N–J tree method (algorism: Poisson correction, bootstrap values: 1000 replicates). Pairwise and multiple alignments were performed with a gap opening penalty of 10 and a gap extension penalty of 0.2. Significant differences were analyzed using GraphPad InStat3 statistical software (GraphPad Software, Inc., San Diego, CA, USA).

## 3. Results

### 3.1. Analysis of cDNA and Deduced Amino Acid Sequence of SfCnc

**Figure 1:**
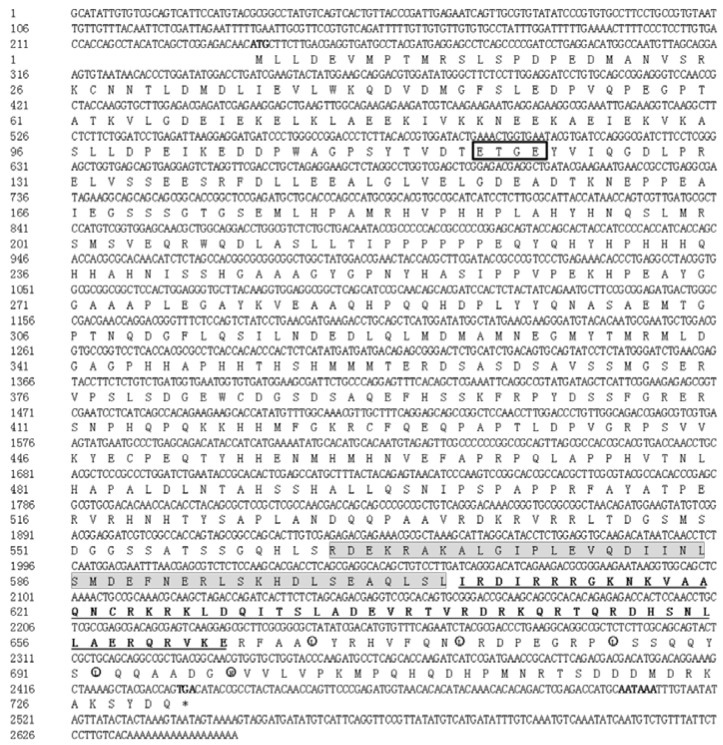
Nucleotide and deduced amino acid sequence of *Sf*CncC. The start codon (ATG), stop codon (TAA), and putative polyadenylation signal (AATAAA) are bond highlighted. The tetrapeptide motif (ETGE) are boxed, amino acids participating in the putative leucine-zipper dimerization domain are circled, the CNC-bZIP DNA binding domain are shown bonded and underlined, and the Cnc homology region (conserved in the NF-E2 family) is shaded.

The cDNA of the *Sf*CncC consists of 2652 nucleotides that include a 2196-nucleotide ORF encoding 731 amino acid residues, and 239- and 217-bp non-coding regions flanking at the 5’- and 3’-ends of the cDNA, respectively. It contains a highly conserved DNA binding domain (CNC-bZIP domain) and a tetrapeptide motif, ETGE, critical for their phylogenetically conserved interaction (Figure 1).

### 3.2. Phylogenetic Analysis of SfCncC

**Figure 2:**
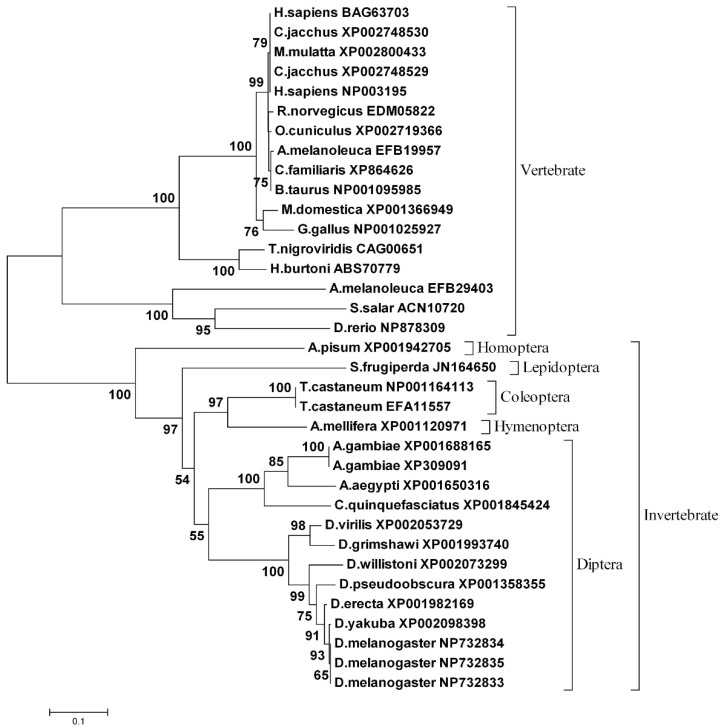
The phylogenetic tree of vertebrate Nrf2 and invertebrate CncC. A non-rooted neighbor-joining tree using Poisson-correction (Bootstrap values: 1000 replicates) distances was constructed with CncC/Nrf2 ORF amino acid sequences of vertebrate and invertebrate species from *Acyrthosiphon pisum*, *Aedes aegypti*, *Ailuropoda melanoleuca*, *Anopheles gambiae*, *Apis mellifera*, *Bos taurus*, *Callithrix jacchus*, *Canis familiaris*, *Culex quinquefasciatus*, *Danio rerio*, *Drosophila erecta*, *Drosophila grimshawi*, *Drosophila melanogaster*, *Drosophila pseudoobscura*, *Drosophila virilis*, *Drosophila willistoni*, *Drosophila yakuba*, *Gallus gallus*, *Haplochromis burtoni*, *Homo sapiens*, *Macaca mulatta*, *Monodelphis domestica*, *Oryctolagus cuniculus*, *Rattus norvegicus*, *Salmo salar*, *Tetraodon nigroviridis*, *Tribolium castaneum*, and *Spodoptera frugiperda*. Genbank accession numbers were given in this figure.

**Figure 3:**
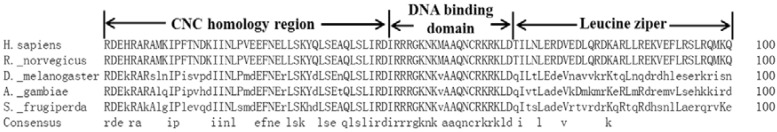
Homology and similarity between nuclear factor erythroid 2 related factor 2 (Nrf2), Cnc isoform C (CncC), and the known members of the NF-E2 family. The conserved regions among the NF-E2 family members are shown in detail. The alignment covers the leucine zipper, the DNA binding domain, and the Cnc homology region immediately N-terminal to the DNA binding domain. Uppercase type, conservative amino acid change; lowercase type, nonconservative amino acid change. (Genbank No.: *Homo sapiens* NP_003195; *Rattus norvegicus* EDM_05822; *Drosophila melanogaster* NP_732833; *Anopheles gambiae* XP_001688165; *Spodoptera frugiperda* JN_164650).

A phylogenetic tree of the full-length CncC (invertebrate) or Nrf2 (vertebrate) was generated by MEGA 6.0 and based on the amino acid sequence alignment with other vertebrate and invertebrate CncC/Nrf2 by using ClustalW. The unrooted tree showed that Cnc/Nrf2 from different species has grouped into vertebrate and invertebrate clusters. CncC from different insect species are clearly grouped into Homoptera, Hymenoptera, Lepidoptera, Diptera, and Coleoptera sub-clusters, and show high identity as compared with the CncC from some other insect species (Figure 2). Compared with the known members of the NF-E2 family, the *Sf*CncC the domain shows conserved regions among the NF-E2 family members. The alignment covers the leucine zipper, the DNA binding domain, and the Cnc homology region immediately N-terminal to the DNA binding domain (Figure 3).

### 3.3. Overexpression Analysis of SfCncC Regulating SOD Gene Transcription in Sf9 Cell

**Figure 4:**
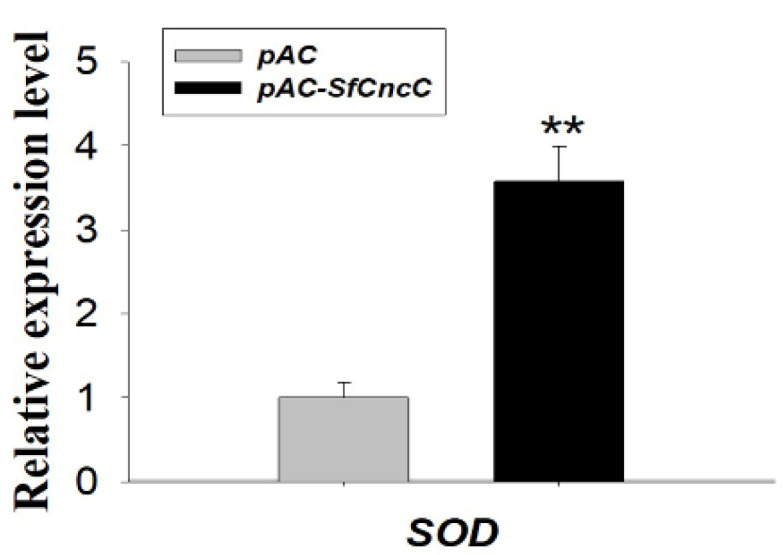
Over-expression analyses of *Sf*CncC regulate SOD gene transcription in Sf9 cells. The mRNA transcription level (means ± SD) of SOD in Sf9 cells transfected with pAC-*Sf*CncC construct and pAC vector. The error bars indicate 95% confidence intervals (n = 3). ** indicated significant difference by Student’s t-text (*p* < 0.01).

Sf9 cells transfected with pAC-*Sf*CncC were harvested after a 96 h incubation at 28 °C for total RNA isolation and cDNA synthesis. Over-expression of *Sf*CncC can significantly up-regulate SOD gene transcription in Sf9 cells, which indicates that *Sf*CncC actually is one of the antioxidant transcriptional factors in *S. frugiperda*, just the same as the function of Nrf2 in vertebrate. The transcription level of SOD from the Sf9 cells transfected with pAC–*Sf*CncC was 3.57-fold higher (*p* < 0.01) than that of cells transfected with a pAC empty vector (Figure 4).

### 3.4. RNAi Analysis of SfCncC Regulating SOD in Sf9 Cell and Larvae

**Figure 5:**
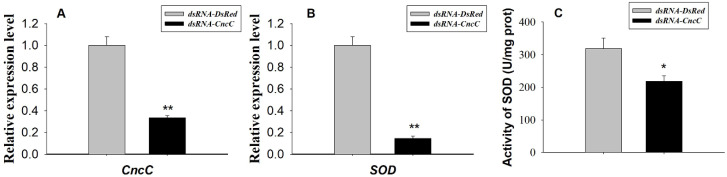
RNAi analyses *Sf*CncC regulate SOD transcriptional levels in Sf9 cells. The mRNA transcription level (means ± SD) of *Sf*CncC (**A**) and SOD (**B**); and the activity of SOD (**C**) in in Sf9 cells transfected with CncC dsRNA and DsRed dsRNA. The error bars indicate 95% confidence intervals (n = 3). * on the columns indicated significant difference by Student’s t-text (*p* < 0.05), ** indicated significant difference by student’s t-text (*p* < 0.01).

**Figure 6:**
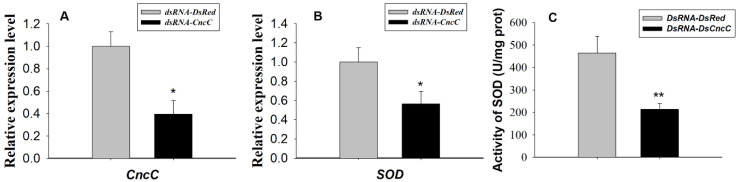
RNAi analyses of *Sf*CncC regulate SOD transcriptional levels and SOD activity in larvae. The mRNA transcription level (means ± SD) of *Sf*CncC (**A**) and SOD (**B**), and activity of SOD (**C**) in larvae injected with CncC dsRNA and DsRed dsRNA (1µg/per larva). The error bars indicate 95% confidence intervals (n = 3). * on the columns indicated significant difference by student’s t-text (*p* < 0.05), ** indicated significant difference by student’s t-text (*p* < 0.01).

The Sf9 cells and larvae transfected/injected with dsRNA (Cnc dsRNA or DsRed dsRNA) were harvested after 96 h or 72 h for total RNA extraction and cDNA synthesis, respectively. Results demonstrated that the knock down of *Sf*CncC leads the significant down-regulated SOD transcription in Sf9 cells (Figure 5). The mRNA transcription level of *Sf*CncC from the Sf9 cells treated with dsRNA-*CncC* was 2.98-fold less (*p* < 0.01) than cells treated with dsRNA-*DsRed* (Figure 5A). The transcription level and activity of SOD from the cells treated with Cnc dsRNA was 6.87- and 1.46-fold less (*p* < 0.01) than cells treated with DsRed dsRNA, respectively (Figure 5B,C). Suppression of SfCncC to 0.39-fold of control also dramatically decreased the transcriptional level of *SOD* to 0.56-fold of control larvae (Figure 6A,B). The RNAi of *SOD* in larvae also reduced the total activity to 0.46-fold of control larvae (Figure 6C).

## 4. Discussion

Transcription factor Nrf2 is a member of the basic leucine-zipper NF-E2 family and interacts with the antioxidant response element (ARE) in the promoter region of protective detoxification and antioxidant genes important in the protection of cells against oxidative damage [1,34,35,36,37]. In our research, we found a 2652 nucleotides length *Sf*CncC cDNA sequence. It consists of a 2196-nucleotide open reading frame (ORF) encoding 731 amino acid residues, and 239- and 217-bp non-coding regions at the 5’- and 3’-ends of the cDNA, respectively (Figure 1). Sequence analysis indicated that it has the conserved domains character of vertebrate Nrf2. Phylogenetic analysis demonstrated that the sequences of *Sf*CncC shares a higher identity when compared with CncC from other insect species (Figure 2 and Figure 3). Detailed analysis of Nrf2 activity and structure revealed that the DNA binding domain CNC-bZIP is an evolutionarily conserved regulatory domain of Nrf2 (Figure 2). The Cnc-bZIP is important for Nrf2 to bind to the ARE DNA sequence of a target gene with small Maf protein as the obligatory partner molecule [5,38]. A tetrapeptide motif, ETGE (Figure 1), which resides close to the C-terminal boundary of Neh2, is critical for phylogenetically conserved interaction [39]. The cytoplasmic actin-binding protein, Keap1, sequesters Nrf2 in the cytoplasm to form a Keap1–Nrf2 complex by binding with the Neh2 domain of Nrf2 [6,11,40]. Once in the cytoplasm, the Keap1–Nrf2 complex associates with the E3 ubiquitin ligase, resulting in the degradation of Nrf2 and the termination of the Nrf2 signaling pathway [41,42]. Inducers, such as chemical oxidants could dissociate this complex allow Nrf2 to translocate to the nucleus [40,43].

In response to stress, Nrf2 is released from Keap1 repression, accumulates in the nucleus, and activates transcription of phase II xenobiotic-metabolizing enzymes genes (e.g., GSTs) and oxidants and their modulating enzymes genes (e.g., SOD). In our experiments, we chose SOD as the target gene for testing the regulation effect of *Sf*CncC. Over-expression of *Sf*CncC in Sf9 cells lead to a greater increase of SOD at the transcriptional level than that recorded in the control cells, as shown by real-time quantitative PCR (Figure 4). In vitro testes have also been done by using RNAi method. Double strand RNA (Cnc dsRNA or DsRed dsRNA) was transfected into Sf9 cells and the expression level of both *Sf*Cnc and SOD have been analyzed after 96 h of transfection. Real-time quantitative RT-PCR results illustrated that the knocking down of the *Sf*CncC gene will dramatically reduce the transcription level and total SOD activity of SOD (Figure 5 and Figure 6), and also reduce the activity of SOD significantly in larvae (Figure 6C). Hence, we can ensure the involvement of *Sf*CncC in the Keap1–Nrf2–ARE pathway acted the same as the transcript factor of Nrf2 in vertebrate, and plays a role for host cell defense. The Keap1–Nrf2–ARE signaling pathway also exists in the *D. melanogaster*, and can be activated by oxidants, induces antioxidant and detoxification responses, and confers increased tolerance to oxidative stress [20,21,29,30,31,32]. Importantly, Keap1 loss-of-function mutations extend the lifespan of Drosophila males, supporting a role for Nrf2 signaling in the regulation of longevity [21]. The clone and functional characterization of *Sf*CncC is the study of the antioxidant regulatory cascades in other agro-destructive insects, expect *D. melanogaster*. *S. frugiperda* is destructive to many crops, however, the question of how they cope with the diversity of xenobiotic chemicals in plant hosts still remains largely a mystery. In vertebrate and insects, a substantial number of genes have been found to contain ARE or related sequences, and can be regulated by Nrf2. Promoters of *GSTo2*, *GSTe6*, and *GSTd3* of *S. exigua* harbor the same CncC/Maf binding site, and were found to be regulated by CncC/Maf and confer resistance to chlorpyrifos and cypermethrin [28]. Some oxidative stress responses in GST, P450, UGT, and NAD(P)H: quinone oxidoreductase have been confirmed to be regulated by Nrf2/CncC [17,18,20,27,29,30,31,32]. The regulatory cascades that provide xenobiotic chemical inducibility still remain largely unknown. The research results in this paper provide some basic information for us to further understand the anti-xenobiotic regulatory mechanism in *S. frugiperda*. Understanding the regulatory cascades will provide us with more insights into the designing of novel and environmentally benign methods to control this pest.

## 5. Conclusions

The present study characterized the Cap ‘n’ Collar Isoform C gene of *S. frugiperda* and its regulation effects on superoxide dismutasea. These results provide some basic information for us to further understand the anti-xenobiotic regulatory mechanism in *S. frugiperda*.
